# Cheap Telescopes

**Published:** 1880-10

**Authors:** 


### CHEAP TELESCOPES.

A writer in *The Scientific American* makes the following remarks on making telescopes in a cheap way:—

The following is a plan which I adopted more than twenty years ago:

I called on a dealer in optical lenses and selected a meniscus, one inch in diameter and of 48 inch focus. This was for my object-glass. I had already in my possession a two-lensed double convex jeweler's eye-glass; one of these lenses was used for the eye-piece, its focal length being a trifle over one inch. The tube was made of pine wood. A piece of straight, evenly grained, one inch pine board, two inches wide and eight feet long, was cut in the middle; and the two pieces, after making a tapering semi-circular groove in each, well glued together. This done, the next thing was to give it a round, tapering form, two inches in diameter at one end and a trifle over an inch at the other. This was done with a common carpenter's plane. I now had a tube four feet long, with a tapering hole through its length, and 1¼ inch in diameter at its largest end. Two wooden cells for the lenses were then turned in a lathe, and were made to go on to the tube, as does the cover of a wooden pill-box. A round hole, the size of the lens, was made in each, the meniscus being contracted to ¾ inch, and the eye-glass to ¼ inch diameter. The piece carrying the eye-glass was made so as to slide some distance on the tube, for adjustment to distant vision. The tube was painted and varnished, and mounted equatorially, and it proved to be a good instrument, showing Jupiter's moons, their movements and eclipses, handsomely; the ring of Saturn, the horned appearance of Venus, the mountains and craters on the moon, the spots on the sun, etc. Several of the nebulæ were also visible, especially those in *Andromeda*, *Orion*, *Hercules* and *Sagittarius*. This instrument has been a great pleasure to me and my family of boys and girls, and even my neighbors, and the whole need not cost over two dollars, besides the time in making, provided one is a mechanic.

The meniscus (concave on one side and convex on the other) is the proper form for a single lens object-glass, and a plano-convex lens makes the best form for the eye-piece. Care must be taken to set the lenses in their cells that their foci will meet centrally. When this is the case the lenses are said to be well centred, and in that way we get rid of most of the prismatic color. Another point that wants attention is the mounting. Absolute steadiness is required for close observation. I used to put mine upon a post set firmly in the ground. The equatorial arrangement for mounting is described in nearly every work on telescopes.

